# Metabolic Benefits vs. Cardiovascular Uncertainty: A Critical Review of GLP-1 Receptor Agonists in Type 1 Diabetes

**DOI:** 10.3390/ijms27093882

**Published:** 2026-04-27

**Authors:** Elżbieta Wójcik-Sosnowska, Adrianna Tabeau, Agnieszka Pawlik, Bartłomiej Węglarz, Leszek Czupryniak

**Affiliations:** Department of Internal Medicine and Diabetology, Medical University of Warsaw, 1a Banacha Street, 02-097 Warsaw, Poland; elzbieta.wojcik-sosnowska@wum.edu.pl (E.W.-S.); adriannatabeau@gmail.com (A.T.); aipawlik01@gmail.com (A.P.); bartek.weglarz@me.com (B.W.)

**Keywords:** type 1 diabetes, GLP-1 receptor agonists, double diabetes, cardiovascular risk, obesity, weight loss

## Abstract

Type 1 diabetes (T1DM) is associated with elevated cardiovascular (CV) risk, often exacerbated by the rising prevalence of obesity. Glucagon-like peptide-1 receptor agonists (GLP-1 RAs) reduce CV risk in type 2 diabetes, but their role in T1DM is less well-defined. This umbrella review synthesizes evidence from systematic reviews, meta-analyses, and Mendelian Randomization (MR) studies to evaluate the metabolic efficacy and safety of GLP-1 RAs in T1DM. Adjunctive therapy, particularly with liraglutide and exenatide, was associated with clinically meaningful weight reduction (mean difference: −4.35 kg to −5.1 kg) and lower total daily insulin doses. HbA1c reductions were statistically significant but modest (0.2–0.3%), with no improvement in Time in Range. Secondary benefits included lower systolic blood pressure. Safety data were mixed: the risk of severe hypoglycemia was not increased, whereas Time Below Range and gastrointestinal adverse events were more frequent. Evidence on diabetic ketoacidosis (DKA) was inconsistent across studies. Overall, GLP-1 RAs improve weight and reduce insulin requirements in T1DM, potentially mitigating indirect CV risk factors; however their direct cardiovascular benefits remain unproven in the absence of dedicated outcome trials.

## 1. Introduction

Type 1 diabetes mellitus (T1DM) is a widespread chronic autoimmune disease, affecting approximately 130,000 people in Poland [1] and 9 million individuals worldwide [2]. The incidence and prevalence of T1DM have been steadily rising, now accounting for 5–10% of all diabetes cases. According to a systematic review and meta-analysis, the global prevalence of T1DM was estimated at 9.5%, with an incidence of 15 per 100,000 people [3]. Furthermore, in Poland, among young adults with T1DM, 35.5% were classified as overweight, and 13.2% were classified as obese, while globally, the latest collective data from 2015 to 2020 indicate that 31.9% of adults with T1DM were overweight, and 22.2% were obese [4].

To ensure prompt and accurate diagnosis of T1DM, it is crucial that healthcare providers possess a comprehensive understanding of the symptoms associated with the condition. Patients exhibiting symptoms such as polyuria, polydipsia, nocturia, and/or unexplained weight loss should undergo diagnostic testing for T1DM. Additional findings associated with T1DM may include abdominal pain, nausea, emesis, blurred vision, lethargy, decreased appetite, and irregular breathing [5].

Diabetes may be diagnosed using criteria relating to plasma glucose, such as fasting plasma glucose or postprandial glucose during a 75 g oral glucose tolerance test (OGTT). Alternatively, the diagnosis may be based on HbA1c levels. The following diagnostic criteria apply:

- Fasting plasma glucose ≥ 126 mg/dL (7.0 mmol/L) on more than one occasion;
- Random plasma glucose ≥ 200 mg/dL (11.1 mmol/L) with classic symptoms of hyperglycemia;
- Plasma glucose ≥ 200 mg/dL (11.1 mmol/L) measured 2 h after a 75 g OGTT;
- HbA1c level ≥ 6.5% [6].

Once the diagnosis of diabetes is confirmed, the specific type must be identified. Differentiation may be achieved through clinical presentation and laboratory studies, including testing for T1DM pancreatic autoantibodies and stimulated C-peptide levels, the latter measuring pancreatic β-cell function. T1DM pancreatic antibodies include ICA, IAA, GAD65, IA-2, and ZnT8. At the time of diagnosis, most patients with T1DM have one or more positive T1DM antibodies [7].

T1DM is associated with various acute and chronic complications. Acute complications include diabetic ketoacidosis and severe hypoglycemia. Chronic complications are categorized into microvascular and macrovascular complications. Microvascular complications include retinopathy, nephropathy, and neuropathy, whereas macrovascular complications involve coronary artery disease and vascular disease [8].

Patients with T1DM exhibit a mortality rate approximately three times higher than that of the general population, contributing to a projected reduction in life expectancy of at least 11 years [9,10]. They also have a 4- to 10-fold increased risk of cardiovascular disease (CVD), with CVD events occurring approximately 10 to 15 years earlier than in individuals without diabetes, especially among women, making CVD one of the leading causes of morbidity and mortality in this population [10,11]. This elevated risk is associated with multiple factors, including elevated mean HbA1c levels, increased systolic blood pressure, higher resting heart rate, elevated triglycerides and LDL cholesterol levels, and longer diabetes duration [11,12]. Although elevated glucose levels have a significant impact, cardiovascular risk remains high even in patients with well-controlled glycemia [10].

Since its discovery in 1921 [13], insulin has remained the cornerstone of treatment for patients with type 1 diabetes [14]. However, despite its life-saving benefits, insulin therapy is not without its challenges. It is associated with various complications, including hypoglycemia, weight gain, and skin reactions at the sites of subcutaneous insulin injections [15,16].

The use of exogenous insulin and intensified insulin therapy are important risk factors for weight gain in patients with type 1 diabetes [17]. Insulin is an anabolic hormone that promotes lipogenesis and inhibits lipolysis, promoting energy storage through preferential deposition of adipose tissue [18]. Chronic hyperinsulinemia leads to increased hunger and food intake. This is often compounded by excessive caloric and carbohydrate consumption, and reduced physical activity owing to fear of hypoglycemia. These effects may lead to insulin resistance, requiring higher insulin doses to maintain glycemic control, thereby perpetuating a cycle of weight gain and metabolic burden. In addition, psychological factors, such as stress and decreased quality of life, further contribute to unhealthy lifestyle behaviors and weight gain in this patient population [16,17,19,20]. This phenomenon has led to the emergence of a clinical phenotype often termed “double diabetes”, in which individuals with type 1 diabetes develop features of insulin resistance and metabolic syndrome typical of type 2 diabetes [16,20]. This condition is frequently driven by the interplay between intensive insulin therapy and weight gain, creating a vicious cycle of increasing insulin requirements and further metabolic deterioration [17,20]. Consequently, patients with “double diabetes” face a disproportionately higher risk of cardiovascular complications and mortality compared to those with classic type 1 diabetes, highlighting the urgent need for adjunctive therapies that address both glycemic control and weight management [16,19].

Glucagon-like peptide-1 receptor agonists (GLP-1 RAs) are a drug class that mimics the action of the naturally occurring hormone GLP-1. These medications are primarily used to treat type 2 diabetes and obesity [21]. GLP-1 RAs enhance the physiological response to nutrient intake by stimulating glucose-dependent insulin secretion, inhibiting glucagon release, slowing gastric emptying and promoting satiety. These combined actions contribute to improved postprandial and overall glycemic control with a low risk of hypoglycemia, facilitating effective long-term glycemic management. In addition, GLP-1 RAs have been associated with weight loss due to reduced appetite and food intake [22,23]. GLP-1 RAs significantly reduce the risk of major adverse cardiovascular events (MACEs), including cardiovascular death, myocardial infarction, and stroke, in patients with and without diabetes. These effects appear to extend beyond improvements in blood pressure, glycemic control, and weight loss. The cardiovascular risk reduction associated with GLP-1 RAs is likely multifactorial and not solely attributable to changes in traditional risk factors [24,25,26,27].

A critical goal in managing type 1 diabetes is to improve the metabolic parameters that drive cardiovascular risk and mortality. The challenge, however, is that intensive insulin therapy can inadvertently lead to a vicious cycle of weight gain and insulin resistance. GLP-1 receptor agonists may hold the key to breaking this cycle. Therefore, the aim of this umbrella review is to synthesize the most recent evidence, including meta-analyses and Mendelian Randomization (MR) studies, to evaluate the metabolic and cardiovascular impact of GLP-1 RAs in adults with T1DM. This dual approach allows us to go beyond observational associations and explore potential causal links. The novelty of this review lies in three principal aspects that distinguish it from previous work. First, it integrates the most recent evidence (2024–2025), including continuous glucose monitoring (CGM) metrics and MR data not covered in earlier reviews [28,29,30,31]. Second, it applies standardized quality-assessment tools (AMSTAR 2 and STROBE-MR) to ensure transparent methodological evaluation [32,33,34]. Third, by combining clinical synthesis with genetic inference, the analysis provides a higher-level insight into causality that traditional narrative reviews cannot capture [30,31,35]. Together, these elements establish a sound scientific and methodological foundation for our ongoing institutional research on the clinical application of GLP-1 RAs in type 1 diabetes.

We hypothesize that improvements in metabolic parameters, such as weight reduction and glycemic stability, may correlate with a reduction in cardiovascular risk markers. By employing rigorous quality assessment using the AMSTAR 2 tool, this review clarifies existing uncertainties regarding long-term outcomes and establishes an evidence-based baseline for future research initiatives at our institution.

## 2. Methods

A systematic literature search was conducted in MEDLINE, Embase, and the Cochrane Library through July 2025. The search utilized keywords and controlled vocabulary (e.g., MeSH) for concepts related to the intervention—such as “GLP-1 receptor agonists,” liraglutide, semaglutide, and dulaglutide—and the population, including “type 1 diabetes” and “LADA”. To ensure maximum sensitivity and minimize the risk of missing relevant evidence, no language restrictions were applied. The search strategy was supplemented by manually screening the reference lists of all included papers. The complete search string used for the MEDLINE database is provided in Appendix A.

To ensure scientific rigor, we applied specific inclusion and exclusion criteria based on the PICO (Population, Intervention, Comparison, Outcome) framework, as detailed in Table 1.

**Figure 1:**
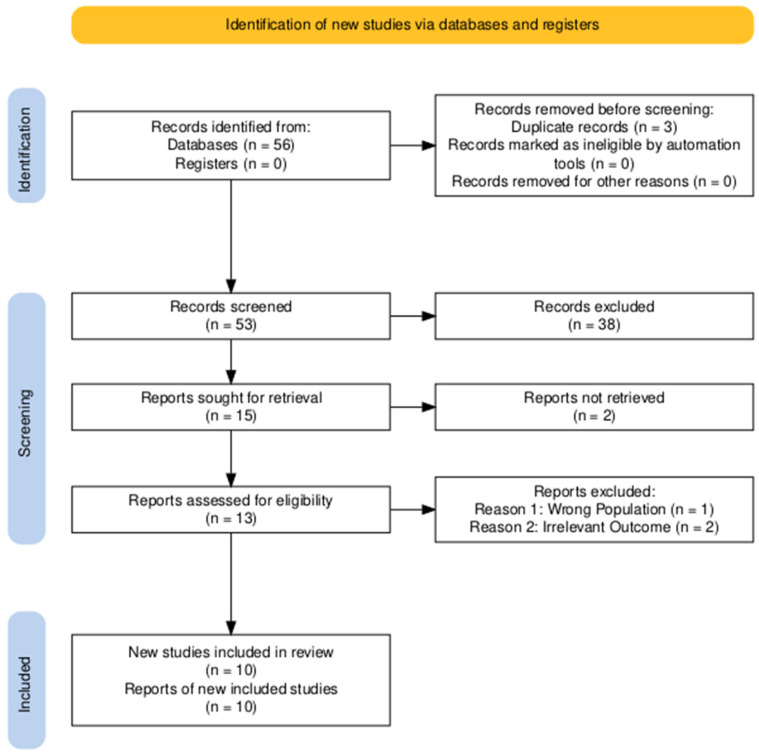
PRISMA 2020 flow diagram.

The selection process was conducted independently by two reviewers, with any discrepancies resolved through consensus or consultation with a third author. The complete process of study identification, screening, and the specific reasons for excluding full-text articles are presented in the PRISMA 2020 flow diagram (Figure 1). Excluded studies are detailed in Table S3.

The methodological quality of the systematic reviews (using AMSTAR 2) and the reporting quality of the Mendelian Randomization studies (using STROBE-MR) were assessed independently by two reviewers. Discrepancies were resolved through consensus or consultation with a third author. The results of these assessments are summarized in Tables S1 and S2, provided in the Supplementary Materials.

Instead of conducting a new statistical meta-analysis, we performed a narrative synthesis, extracting key data on study design, population, and conclusions. These findings were then grouped by themes—such as glycemic control, weight management, and safety—to qualitatively identify patterns and gaps in the evidence.

Due to the nature of this work being an umbrella review, a formal statistical assessment of publication bias was not conducted. Instead, where available, we relied on the assessments provided by the authors of the included systematic reviews. This limitation is acknowledged and discussed in the limitations section.

## 3. Results and Discussion

### 3.1. Efficacy Outcomes

The primary benefits of adjunctive GLP-1 RA therapy in T1DM appear to be metabolic rather than glycemic, with a consistent and dose-dependent reduction in body weight as the most significant finding [32,33,34,36,37]. Quantitative results regarding weight, HbA1c, and insulin dose reduction from the included meta-analyses are summarized chronologically in Table 2.

As shown in the synthesized data, liraglutide 1.8 mg was associated with a mean weight loss of 4.89 kg (95% CI: −5.33 to −4.45) [32], a finding corroborated by other comprehensive reviews that reported nearly identical reductions [33,36]. The benefits extend to other agents; for instance, exenatide demonstrated significant weight loss across separate network meta-analyses [34,37]. Interestingly, one of these analyses did not find a similar significant effect for liraglutide, in contrast to the broader literature [37]. This impact on weight is complemented by improvements in other cardiovascular risk factors, such as a significant reduction in systolic blood pressure of −3.39 mmHg (95% CI: −4.37 to −2.41) with liraglutide [34], whereas another review noted significant decreases in systolic and/or diastolic blood pressure in several included studies [36].

A consistent insulin-sparing effect is also a well-supported finding across the reviewed studies [28,32,33,34,36]. Notable reductions in total daily insulin requirements were observed for both exenatide (−10.23 IU; 95% CI: −15.78 to −4.68) [34] and liraglutide (−7.51 IU; 95% CI: −9.52 to −5.49) [32].

In contrast, the direct impact on glycemic control is modest, with findings that are not entirely consistent across the literature. Most meta-analyses report a small but statistically significant reduction in HbA1c (typically ranging from −0.21% to −0.28%) [28,29,32]. However, these findings were not uniform; one network meta-analysis observed this benefit for liraglutide (95% CI: −0.34 to −0.11) but not for daily exenatide [34], while another found no statistically significant reduction for either agent [37]. Furthermore, this modest HbA1c improvement does not appear to translate into better glycemic stability. A critical insight comes from a meta-analysis that found that GLP-1 RA therapy failed to improve Time in Range, showing a non-significant mean difference of −0.22% (95% CI: −2.39 to 1.95), and was associated with a significant increase in Time Below Range of 1.13% (95% CI: 0.50 to 1.76). The authors critically note that these findings are based on older, short-acting agents, and their applicability to newer medications remains unknown [29].

From a pharmacodynamic perspective, GLP-1 receptor agonists enhance glucose-dependent insulin secretion, suppress glucagon release, delay gastric emptying, and promote satiety [21,22]. These combined effects contribute to improved overall metabolic control without increasing the intrinsic risk of hypoglycemia [23].

The pharmacokinetic characteristics of individual agents partly explain observed differences in efficacy. Longer-acting compounds such as semaglutide or dulaglutide ensure more sustained receptor engagement, whereas shorter-acting agents like exenatide are associated with greater gastrointestinal variability [21,24]. Such distinctions should be considered when interpreting cross-study results, as pharmacokinetic half-life and receptor affinity influence both weight reduction and insulin-sparing effects [22,23]. These pharmacologic determinants correspond with the consistent reductions in weight and insulin requirements observed across meta-analyses (Table 2), particularly with longer-acting agents such as semaglutide and dulaglutide.

### 3.2. Safety Outcomes

From a safety perspective, the therapy presents a mixed profile. Reassuringly, adjunctive GLP-1 RA therapy did not appear to increase the risk of severe hypoglycemic events (OR 0.67; 95% CI, 0.43–1.04) [32]. This is contrasted, however, by CGM data suggesting a higher frequency of non-severe hypoglycemic events, as evidenced by an increased Time Below Range (MD = 1.13%, 95% CI: 0.50–1.76) [29]. More pressing safety concerns include a heightened risk of ketosis and a high incidence of gastrointestinal side effects. Liraglutide use was associated with significantly higher odds of both ketosis (OR 1.8; 95% CI: 1.1–2.8) and nausea (OR 6.5, 95% CI: 5.0–8.4) [32], often accompanied by vomiting and diarrhea, with these gastrointestinal symptoms being a primary driver of treatment discontinuation [32,33]. However, evidence regarding ketoacidosis remains conflicting; other network meta-analyses did not find a statistically significant increase in the risk of diabetic ketoacidosis (DKA) or serious adverse events (SAEs) for GLP-1 RAs compared to placebo [29,33,34,37]. Beyond adverse event profiles, drug safety also depends on pharmacokinetic and pharmacogenomic factors [21,24]. GLP-1 receptor agonists are degraded primarily by endogenous endopeptidases rather than hepatic cytochromes, minimizing pharmacokinetic interactions [21]. However, interindividual genetic differences in GLP1R expression and downstream signaling may influence tolerability, particularly regarding gastrointestinal side effects [24,30]. Current evidence in type 1 diabetes remains scarce, underscoring the need for dedicated pharmacogenomic and pharmacokinetic studies to optimize dosing and safety monitoring. In clinical practice, discontinuation due to adverse events occurred more frequently with GLP-1 RA therapy than with placebo, highlighting the clinical importance of gradual dose titration and careful patient selection [32,33].

### 3.3. Evidence from Genetic Studies and Immunomodulatory Pathways

GLP-1 receptor agonists exert diverse biological effects that extend beyond glycemic control. Preclinical data demonstrate anti-inflammatory and β-cell-protective actions via cytokine-signaling inhibition and reduction in oxidative stress [35]. These mechanisms may contribute to autoimmune modulation in T1DM [30].

From a pharmacogenomic and receptor-level standpoint, variability in GLP1R gene expression and post-receptor signaling appears to underlie interindividual differences in metabolic response [24,31]. Population-based analyses indicate that common single-nucleotide variants within GLP1R may affect responsiveness to therapy, though data specific to T1DM remain limited.

Recent Mendelian Randomization studies highlight the biological complexity of this pathway: one showed a protective association between genetically proxied GLP1R activation and lower T1DM risk (OR = 0.76; 95% CI 0.67–0.87) [30], whereas another study revealed an opposite effect with a 28% increased risk of developing the disease (OR 1.28, 95% CI: 1.13–1.44) [31]. These contrasting findings suggest that genetic and immunologic context influences how GLP-1 signaling affects disease risk and response.

Integrating pharmacodynamic, pharmacokinetic, and genetic evidence supports a multifactorial framework for understanding the clinical effects of GLP-1 RAs in T1DM [21,30,31,35].

### 3.4. General Discussion and Future Perspectives

Patients with type 1 diabetes (T1DM) face a significantly elevated risk of cardiovascular disease, which is a leading cause of premature mortality in this population [9,10]. This risk is driven not only by hyperglycemia but also by a high prevalence of traditional cardiovascular risk factors, including obesity and hypertension, often exacerbated by the metabolic consequences of intensive insulin therapy, such as weight gain and insulin resistance [16,17].

The potential mechanism linking GLP-1 RA use to cardiovascular risk reduction in T1DM differs fundamentally from T2DM. In T1DM, the primary driver of cardiovascular risk is often the “double diabetes” phenotype—a vicious cycle in which intensive insulin therapy leads to weight gain and insulin resistance. GLP-1 RAs may break this cycle by suppressing inappropriate postprandial glucagon secretion and delaying gastric emptying. This enables a reduction in total daily insulin dose despite improved metabolic control, directly targeting visceral adiposity. This review sought to determine whether adjunctive GLP-1 receptor agonist (GLP-1 RAs) therapy could address these metabolic challenges. Our synthesis of the current evidence reveals that while direct proof of cardiovascular protection is still lacking, these agents consistently and substantially improve key surrogate markers for cardiovascular risk.

The most robust finding of this review is the significant and dose-dependent reduction in body weight, a major modifiable risk factor for cardiovascular disease. This conclusion is strongly supported by high-quality evidence from the meta-analysis by Park et al. [32], which indicates a mean weight loss of nearly 5 kg with higher doses of liraglutide. Although other reviews with critically low methodological ratings also reported similar quantitative effects [33,36], their flaws indicate that the findings from Park et al. should be considered the primary evidence base. This benefit is complemented by a significant reduction in systolic blood pressure and a consistent insulin-sparing effect, which may help break the cycle of weight gain and increasing insulin requirements [32,33,34].

These findings regarding weight loss and reduced insulin requirements are particularly relevant for the “double diabetes” phenotype. GLP-1 RAs appear to directly target the underlying insulin resistance driven by obesity and intensive insulin regimens. Breaking this vicious cycle is critical, as it may positively influence the long-term cardiovascular trajectory—a risk known to be disproportionately high in patients presenting features of both type 1 and type 2 diabetes [16,19,20].

In contrast, the impact on glycemic control is less clear. While high-quality evidence confirms a modest improvement in HbA1c [32], this does not appear to translate into better glycemic stability. A critical insight comes from the CGM data from Karakasis et al. [29], which, despite its “Low” AMSTAR 2 rating, found no improvement in Time in Range and an increase in time spent in mild hypoglycemia.

The clinical utility of GLP-1 RAs is further complicated by a mixed safety profile. Reassuringly, the conclusion that the therapy does not increase the risk of severe hypoglycemia is a robust finding, supported by high-quality evidence [32]. However, this must be weighed against a heightened risk of ketosis [32] as well as a high incidence of gastrointestinal side effects, which are a major driver of treatment discontinuation [32,33].

The underlying biological role of the GLP-1 pathway in T1DM also remains ambiguous. As detailed in our quality assessment (Table S2), two methodologically robust, high-quality Mendelian Randomization studies presented fundamentally contradictory conclusions—one suggesting a protective effect against disease development, and the other an increased risk [30,31].

Although clear metabolic benefits are evident, the pharmacologic diversity among GLP-1 receptor agonists warrants attention [21,24]. Differences in half-life, molecular size, and receptor affinity may partly explain variability in metabolic and safety outcomes, yet the overall class trend supports a shared incretin-mimetic mechanism [22,23]. Whether these pharmacological distinctions translate into measurable differences in long-term cardiovascular outcomes remains to be determined [24,25,26]. Genetic and pharmacogenomic profiling may eventually help identify patients most likely to benefit from adjunctive GLP-1 RA therapy in T1DM [30].

The most significant limitation across all the reviewed literature is the complete absence of long-term cardiovascular outcome trials (CVOTs) for GLP-1 RAs in the T1DM population. This evidence gap, highlighted by nearly every reviewed author, means that any discussion of cardiovascular protection remains based on indirect evidence. Furthermore, as critically noted by Karakasis et al. [29], most available data are derived from older, short-acting agents, and the applicability to newer, more potent medications like semaglutide remains unknown.

In conclusion, GLP-1 RAs offer a promising strategy for improving metabolic health and the cardiovascular risk profile of adults with T1DM, primarily through significant weight reduction. However, the decision to use this therapy requires a careful balancing of these benefits against the clear risks and the current lack of direct evidence for cardiovascular protection. Future research, particularly dedicated CVOTs with newer agents, is essential to definitively establish the role of GLP-1 RAs in improving cardiovascular health and longevity in this population.

## 4. Conclusions

Adjunctive GLP-1 RA therapy offers significant metabolic benefits for adults with T1DM, most notably through substantial and consistent weight reduction. By addressing key cardiovascular risk factors such as obesity and hypertension, these agents represent a promising strategy for improving the overall metabolic health of this high-risk population. However, these benefits must be carefully weighed against a significant burden of gastrointestinal side effects, a heightened risk of ketosis, and a complex effect on glycemic control that does not demonstrate improvement in Time in Range.

Critically, there is a complete absence of long-term data from cardiovascular outcome trials, meaning any potential for reducing cardiovascular events and improving longevity remains unproven. Therefore, while GLP-1 RAs are a valuable tool for metabolic management in select patients with T1DM, future research, particularly dedicated CVOTs with newer agents, is essential to definitively establish their role in cardiovascular protection.

## Figures and Tables

**Table 1:** Inclusion and exclusion criteria based on the PICO framework.

Criterion	Inclusion Criteria	Exclusion Criteria
Population (P)	Adults (≥18 years) diagnosed with type 1 diabetes (T1DM)	Children, adolescents, patients with type 2 diabetes, pregnant women
Intervention (I)	GLP-1 receptor agonists (e.g., liraglutide, semaglutide) as adjunctive therapy	Other non-insulin agents (SGLT2i, Metformin) as the primary focus
Comparator (C)	Standard insulin therapy or placebo	Lack of a control group
Outcomes (O)	Weight change, HbA1c, CGM metrics (e.g., TIR), CV risk markers, safety (DKA)	Studies not reporting metabolic or cardiovascular outcomes
Study Type (S)	Systematic reviews with meta-analyses, Mendelian Randomization (MR) studies	Case reports, animal studies, narrative reviews without meta-analysis, letters

Abbreviations: Lira: liraglutide; Sema: semaglutide; Exe: exenatide; Dula: dulaglutide; Albi: albiglutide; Lixi: lixisenatide; MD: mean difference; SMD: Standardized Mean Difference; CI: Confidence Interval (or Credible Interval for Network Meta-Analyses); Pooled GLP-1 RAs: analysis including all identified agonists (e.g., Lira, Exe, Sema, Dula, Albi, Lixi, Taspoglutide). * Data specifically for liraglutide 1.8 mg.

**Table 2:** Summary of metabolic and glycemic efficacy outcomes (weight, HbA1c, and insulin dose) from included systematic reviews and meta-analyses.

Study (Year)	GLP-1 RAs	Weight Change (MD [95% CI])	HbA1c Change (MD [95% CI])	Insulin Dose Reduction (MD [95% CI])
Alhowiti (2025) [28]	Lira, Exe, Albi	—	SMD 0.23 [0.14, 0.32]	Significant reduction
Karakasis (2024) [29]	Lira, Exe, Sema, Dula, Lixi	—	−0.21% [−0.36, −0.06]	—
Park (2023) [32]	Lira, Exe, Albi	−4.89 kg [−5.33, −4.45] (Lira) * −4.06 kg [−5.33, −2.79] (Exe) +0.08 kg [−2.06, +2.22] (Albi)	−0.28% [−0.38, −0.19] (Lira) * −0.17% [−0.28 to 0.02] (Exe) +0.10% [−0.77, +0.97] (Albi)	−7.51 IU [−9.52, −5.49] (Lira) * −9.76 IU [−15.59, −3.92] (Exe) +5.29 IU [−3.80, +14.38] (Albi)
Avgerinos (2021) [34]	Lira, Exe,	−4.35 kg [–5.53, −3.17] (Exe) −3.85 kg [−4.35, −3.35] (Lira)	−0.23% [−0.34, −0.11] (Lira)	−10.23 IU [−15.7, −4.68] (Exe) −6.61 IU [−8.7, −4.48] (Lira)
Cai (2021) [33]	Pooled GLP-1 RAs	−4.76 kg [−4.95, −4.57]	−0.19% [−0.29, −0.1]	−5.53 IU [−7.79, −3.28]
Tandon (2021) [36]	Lira, Exe	−4.85 kg [−5.29, −4.41] (Lira) * Significant reduction (Exe)	Significant reduction (Lira)	Significant reduction
Kim (2020) [37]	Lira, Exe	−5.1 kg [−8.4, −2.0] (Exe) Non-significant (Lira)	Non-significant	Non-significant

## Data Availability

No new data were created or analyzed in this study. Data sharing is not applicable to this article.

## Supplementary Materials

The following supporting information can be downloaded at https://www.mdpi.com/article/10.3390/ijms27093882/s1, Reference [38] is cited in the supplementary materials.

## Appendix A. Search Strategy

**Database**	**Date of Search and Filters**	**Search Details (Query)**
MEDLINE (via PubMed)	Date: 31 July 2025	( ("glucagon-like peptide 1 receptor agonists" OR "glp-1 agonists" OR dulaglutide OR semaglutide OR liraglutide OR exenatide OR lixisenatide OR tirzepatide) AND ("type 1 diabetes" OR t1dm OR "diabetes mellitus type 1" OR "latent autoimmune diabetes" OR lada) ) AND ("2015/01/01"[Date - Publication] : "2025/07/31"[Date - Publication])
	Timeframe: 01/01/2015-31/07/2025–	
	Filters:Article Type:Meta-Analysis	
Cochrane Library (CDSR—Cochrane Database of Systematic Reviews)	Date: 31 July 2025	(("glucagon-like peptide 1 receptor agonists" OR dulaglutide OR "glp-1 agonists" OR semaglutide OR liraglutide)) AND (("type 1 diabetes" OR t1dm OR diabetes mellitus type 1) OR ("latent autoune diabetes in adults" OR lada))
	Filters: Cochrane Reviews	
Embase (via Elsevier)	Date: 31 July 2025	(2015:py OR 2016:py OR 2017:py OR 2018:py OR 2019:py OR 2020:py OR 2021:py OR 2022:py OR 2023:py OR 2024:py OR 2025:py) AND ([adult]/lim OR [aged]/lim OR [middle aged]/lim OR [very elderly]/lim OR [young adult]/lim) AND (’meta analysis’/de OR ’mendelian randomization’:ti,ab OR ’genetic instrument’:ti,ab OR ’mendelian randomization analysis’:ti,ab) AND (’type 1 diabetes mellitus’/exp OR ’latent autoimmune diabetes’/exp OR ’type 1 diabetes’:ti,ab OR t1dm:ti,ab OR lada:ti,ab OR ’latent autoimmune diabetes’:ti,ab) AND (’glucagon like peptide 1 receptor agonist’/exp OR ’liraglutide’/exp OR ’semaglutide’/exp OR ’dulaglutide’/exp OR ’exenatide’/exp OR ’lixisenatide’/exp OR ’glp-1 agonist*’:ti,ab OR liraglutide:ti,ab OR semaglutide:ti,ab OR dulaglutide:ti,ab OR exenatide:ti,ab OR lixisenatide:ti,ab)
	Filters:Years: 2015–2025, Age: Adult/Elderly, Types: Meta-analysis, Genetic instru-ments	
