# Supplementary material for: Metabolic Benefits vs. Cardiovascular Uncertainty: A Critical Review of GLP-1 Receptor Agonists in Type 1 Diabetes
Source: Int J Mol Sci. 2026 Apr 27;27(9):3882. doi: 10.3390/ijms27093882 (PMC13164057; doi:10.3390/ijms27093882)
Supplement: Supplementary file 1 [file ijms-27-03882-s001.zip › ijms-4102859-supplementary S1-S3.pdf]

Table S1. Methodological Quality Assessment of Included Systematic Reviews (AMSTAR 2).

Study (Author, Year)	AMSTAR 2 Quality Rating	Summary of Flaws & Relevance for this Review
**Karakasis et al. (2024)**	Low	**Relevance:** A contemporary meta-analysis providing crucial CGM data. **Flaws:** Its low formal quality, including no assessment of publication bias (Q15), requires its conclusions to be cited with caution.
**Tandon et al. (2021)**	Critically Low	**Relevance:** Key source for quantitative data on weight reduction (especially liraglutide). **Flaws:** Must be cited with caution due to serious flaws, including no list of excluded studies (Q7), RoB not assessed in duplicate (Q9), and no assessment of publication bias (Q15).
**Cai et al. (2021)**	Critically Low	**Relevance:** Provides valuable numerical data for the GLP-1 RAs subgroup (HbA1c, weight, insulin). **Flaws:** Cautious citation is required due to methodological issues like no list of excluded studies (Q7) and no interpretation of RoB's impact (Q13).
**Wu et al. (2021)**	Critically Low	**Relevance:** Primarily valuable for its 'Discussion' section on potential immunomodulatory mechanisms. **Flaws:** Numerous critical flaws (Q2, Q7, Q9, Q13, Q15) limit the reliability of its primary analysis.
**Alhowiti & Mirghani (2025)**	Critically Low	**Relevance:** Serves as an example to illustrate the problem of low-quality publications in the field. **Flaws:** Contains multiple critical weaknesses across the board (Q2, Q4, Q7, Q9, Q13, Q15).
**Park et al. (2023)**	Low (formally) / High (substantively)	**Relevance:** An excellent, high-quality source for efficacy (weight, HbA1c, insulin) and safety data (no increased risk of severe hypoglycemia). **Flaws:** The only minor weakness is the lack of a pre-registered protocol (Q2).
**Avgerinos et al. (2021)**	Critically Low	**Relevance:** A valuable network meta-analysis for data on CV risk factors (weight, BP) and safety. **Flaws:** Limited by an English-only search (Q4) and no list of excluded studies (Q7).
**Kim et al. (2020)**	Critically Low	**Relevance:** Interesting network meta-analysis showing a strong effect of exenatide on body weight. **Flaws:** Must be used with great caution due to multiple flaws, including no protocol (Q2) and no assessment of publication bias (Q15).

Table S2. Methodological Quality Assessment of Mendelian Randomization Studies (STROBE-MR).

Study (Author, Year)	Quality Rating (STROBE-MR)	Main Finding & Relevance for this Review
**Yang et al. (2025)**	High	**Finding:** Increased *GLP1R* gene expression has a causal, **protective effect** on the risk of developing T1DM. **Relevance:** Provides strong genetic evidence supporting a beneficial, disease-modifying hypothesis.
**Sun et al. (2025)**	High	**Finding:** Increased *GLP1R* gene expression is associated with an **increased risk** of developing T1DM (OR 1.34). **Relevance:** Contradicts Yang et al., which is extremely valuable for discussion, showing the ambiguity of current genetic evidence.

Table S3. List of Excluded Studies at Full-Text Assessment Stage.

ID	Author(s) and Year	Title of Article (Fragment)	Reason for Exclusion (Code)
**1**	Dimitrios P, et al. (2020)	Liraglutide as Adjunct to Insulin Treatment in Patients with Type 1 Diabetes...	Full Text Unavailable (5)
**2**	Clement Lo, et al. (2018)	Insulin and glucose-lowering agents for treating people with diabetes and chronic kidney disease.	Inappropriate Disease (4)
**3**	Rizos et al. (2024)	The effect of SGLT2 inhibitors and GLP1 receptor agonists on arterial stiffness...	Other/Irrelevant to primary objective (6)
**4**	Greco et al. (2022)	Effect of the Glucagon-Like Peptide-1 Receptor Agonists on Autonomic Function...	Other/Irrelevant to primary objective (6)
**5**	Lin et al. (2022)	Efficacy and safety of liraglutide for obesity and people who are overweight...	Full Text Unavailable (5)

Legend of Exclusion Criteria

Code	Exclusion Criterion	Detailed Rationale
**1**	Inappropriate Study Type	The article is a primary study (e.g., Clinical Trial) or a different type of secondary study (e.g., Narrative Review), and is not a Systematic Review with Meta-analysis or a Mendelian Randomization study.
**2**	Inappropriate Intervention	The study focuses on drug classes other than Glucagon-like peptide-1 receptor agonists (GLP-1 RAs).
**3**	Inappropriate Population/Age	The study focuses on a pediatric population (<18 years).
**4**	Inappropriate Disease	The study focuses on Type 2 Diabetes Mellitus (T2DM) or other diabetes types, which is outside the scope of the review.
**5**	Full Text Unavailable	The full text of the article (including meeting abstracts and proceedings) could not be obtained, or the available material was insufficient for full quality assessment.
